# Structure/epitope analysis and IgE binding activities of three cyclophilin family proteins from *Dermatophagoides pteronyssinus*

**DOI:** 10.1038/s41598-023-40720-6

**Published:** 2023-08-21

**Authors:** Yuwei Li, Xizhuo Sun, Liteng Yang

**Affiliations:** 1 Department of Respiratory Medicine, The Seventh Affiliated Hospital of Southern Medical University, Foshan, 528244 Guangdong China; 2 Department of Respirology and Allergy, The Third Affiliated Hospital of Shenzhen University, Shenzhen, 518020 China; 3 Laboratory of Chemical Biology and State Key Laboratory of Rare Earth Resource Utilization, Changchun Institute of Applied Chemistry, Chinese Academy of Sciences, Changchun, 130022 Jilin China

**Keywords:** Cell biology, Immunology, Molecular biology, Molecular medicine

## Abstract

Cyclophilins (CyPs) are involved in basic cellular functions and a wide variety of pathophysiological processes. Many CyPs have been identified as the aetiological agent and influence on the immune system. In the present study, the physicochemical and immunologic characteristics of three proteins of CyPs family (CyPA, CyPB and CyPE) were analyzed. The results indicated that CyPE showed a closer evolutionary relationship with allergenic CyPA. The structure and antigenicity of CyPE was significantly similar with CyPA. B-cell epitopes of CyPE and CyPA were predicted via multiple immunoinformatics tools. Three consensus B-cell epitopes of CyPE and CyPAs were finally determined. To verify results of in silico analysis, three proteins of CyPs family (CyPA, CyPE and CyPB) were cloned and expressed from *Dermatophagoides pteronyssinus*. ELISA results indicated that the positive reaction rates of the three proteins to patient serum are CyPA (21.4%), CyPE (7.1%), and CyPB (0%), illustrating that the IgE activity was exhibited in CypA and CypE excluding CyPB. Structure and immunoinformatics analysis demonstrated that the RNA-binding motif of CyPE could reduce the immunogenicity of PPIase domain of CyPE. The reason that CyPB has no IgE activity might be the structure mutation of CyPB on B-cell epitopes.

## Introduction

Cyclophilins (CyPs) are well-known pan-allergens and have universal ranging cross-reactivity^1,2^, playing very important roles in IgE-mediated poly-sensitization^3,4^. CyP, a peptidyl-prolyl cis/trans isomerase (PPIase, EC 5.2.1.8)^5^, has crucial roles in many physiological and pathological processes, including protein folding and trafficking^6,7^, immune and infectious diseases^8^, inflammation^9^, and malignant tumors^10^. CyP has been reported to be the major intracellular receptor for cyclosporin A (CsA)^11,12^, which is one of the most important immunosuppressant drugs used for prevention of graft rejection after transplant surgery^12,13^. Binding of the CsA-Cyp complex to calcineurin inhibits the phosphatase activity of calcineurin^14^, preventing the translocation of nuclear factor of activated T cells (NF-AT) from the cytosol to the nucleus leading to attenuate the T cell-mediated graft rejection^15,16^. The native CyPs act as autoallergen and cross-react with environmental cyclophilins in atopic dermatitis patients. Thus, the epitope mimicry and new IgE binding of CyPs is conducive to expand understanding of the CyPs mediated physiological and pathological processes.

CyPs have been identified with five classic CyP isoforms (CyPA, B, C, D and E)^11,17^. CyPs are found in all cells of all organisms studied including mammals, plants, insects, fungi, and bacteria^18^. CyPA was reported as a main allergen. Pci c 2 (from *Psilocybe cubensis*) ^19^, Asp f 11 (from *Aspergillus fumigatus*)^20^, Mal f 6 (from *Malassezia furfur*)^21^, Bet v 7 (from *Betula verrucosa*)^22^, and Cat r 1 (from *Catharanthus roseus*)^23^ have been recognized as IgE-binding proteins^24,25^. The house dust mites (HDM) are an important source of indoor allergens that contribute to the rising incidence of allergic sensitization and disease manifestations such as allergic bronchial asthma, perennial allergic rhinitis, and atopic dermatitis^26–28^. More than 30 groups of allergenic components have been identified^29^. CyPs of HDM may play an important role in the mite-induced atopic illnesses^30^.

In the present study, we cloned three Cyp isoforms (CyPs) identified from *D. pteronyssinus*, which are named CyPA, CyPB, and CyPE. We analyze the immunogenicity of CyPs and identify the B-epitopes of CyPs using in silico approaches. These findings provide theoretical support for mite allergen peptide-based vaccine design.

## Materials and methods

### Materials

Trizol was obtained from Beyotime (Shanghai, China) for extracting total RNA. BeyoRT™ First Strand cDNA Synthesis Kit was purchased from Beyotime (Shanghai, China). Primers were synthesized by Sangon Biotech Limited Company (Shanghai, China). *E. coli* Rosetta-gami (DE3) containing pET32a^+^ obtained from Gene Denovo Biotechnology Co. (Guangzhou, China). The antibodies were used for detecting IgE from Thermo Fisher (Waltham, MA, USA) (77,161). Thetetramethylbenzidine (TMB) was obtained from Sigma-Aldrich (St Louis, MO, USA). Other chemicals were all analytical grade and purchased from Sigma-Aldrich (St. Louis, MO, USA).

### Phylogenetic analysis and homology modeling

The phylogenetic tree and the sequence alignment was carried out by MEGA and Clustal X. Homologous protein sequences were obtained from NCBI website^31^. The physicochemical characteristics of CyPs are predicted and analyzed by ProtParam^32^. The secondary structure of CyPs was predicted and analyzed by PSIPRED^33^. Homology modeling was performed through using the best template on the SWISS-MODEL web server^34^. PROCHECK^35^, ERRAT^36^, QMEAN^37^, Ramachandran diagram^38^, ProSA^39^ and VERIFY^39^ program were used to check the original structure model for identifying the correct structure^40^.

### In silico prediction of epitopes

Cbtope^41^, BCPred^42^, DNAStar protean system^43^ and BepiPred 1.0 server^44^ were used to predict B-cell epitopes of CyPs. According to a previously published method, the consensus epitopes of the four tools predicting results were considered to be the ultimate predicting results of B-cell epitopes^45^. Cbtope predicted B-cell epitopes through using an artificial neural network in antigen sequences^41^. BCPred, DNAStar protean system, and BepiPred 1.0 server just need the amino acid sequence and provide more straightforward results^46^.

### Cloning, expression, purification of HDM CyPs

The gene sequence of CyPA (GenBank accession number: ATI08942.1), CyPB (GenBank accession number: XP_027196920.1), and CyPE (GenBank accession number: XP_027199043.1) was obtained by blast with the whole genome of *D. pteronyssinus*, respectively. Total RNA was extracted from the homogenate of *D. pteronyssinus* using Trizol (Beyotime, Shanghai, China) in accordance with the manufacturer's recommendations. The cDNA was synthesized with BeyoRT™ First Strand cDNA Synthesis Kit (Beyotime, Shanghai, China) following the manufacturer’s protocol. Three pairs of specific primers were designed as follows: CyPA primers: 5′-CGGATCCATGGCACTTCCGCGTGTTTA-3′ (Forward, BamH I) and 5′-GGAATTC AAGTTGACCACAATCAGAAAT-3′ (Reserve, Eco RI), CyPB primers: 5′-CGGATCCATGATTGTTCTTATCACCAT-3′ (Forward, BamH I) and 5′-GGAATTCTCGTTAATGGCACACCATTA-3′ (Reserve, Eco RI), CyPE primers: 5′-CGGATCCATGATCGAAATTTCAATCGGCCTGA-3′ (Forward, BamH I) and 5’-GGAATTC ACAAAAATTATTAAATAA-3′ (Reserve, Eco RI). The cDNA was used as the template for PCR and the acquired products were subcloned into the pET32a^+^ expression vector and expressed E. coli Rosetta-gami (DE3). *E. coli* Rosetta-gami (DE3) was cultured at 30 °C. 0.1 mM IPTG was added after the OD 600 nm value is 0.8. The produced proteins (CyPA, CyPB and CyPE) were soluble in supernatant of crude extract. The recombinant CyPs, containing the C-terminal polyhistidine (6 × His) tag, were further purified by affinity chromoatography. Endotoxin was removed using ToxinEraser Endotoxin Removal Kit (L00338, Genscript, China). After checking the LPS concentration value with the Chromogenic LAL Endotoxin Assay Kit (L00350C, Genscript, China), the concentration values are less than 0.1μEU/ml. Proteins were analyzed according to 12% SDS-PAGE colored with Coomassie brilliant blue. The recombinant proteins were collected and stored at 4 °C.

### ELISA for analysis of IgE reactivity

The serums of patients with dust-mite allergic disease were obtained from the First Affiliated Hospital of Guangzhou Medical University. The serums from non-allergic persons were used as control. Each serum sample was provided with a written informed consent from the participant. The Human Ethic Committee at Shenzhen University (Shenzhen, Guangdong, China) approved the use of human sera. All methods were carried out in accordance with relevant guidelines and regulations or Declaration Helsinki in the present study. The antigen-specific IgE in patient serum to CyPs (CyPA, CyPB and CyPE) were detected by indirect ELISA. In short, the 96-well plate was coated at 4 °C for 12 h with 100 μL of compound sample that consists of phosphate buffered saline (20 mM, pH7.0) including CyPA (50 ug/ml) or CyPB (50 ug/ml) or CyPE (50 ug/ml). Subsequently, 200 μL of 3% BSA in PBS was blocked at 37 °C for 2 h. Allergic serums to dust mites were diluted to five times and incubated at 4 °C for 12 h. A peroxidase-labeled mouse anti-human IgE (1:2,000) monoclonal antibody was used to incubate the plates at 37 °C for 1 h. Then the plates were washed with PBST (phosphate buffered saline containing Tween-20) six times. Color the plate and stop with 2 M H_2_SO_4_. The OD values at 450 nm were measured on a microplate reader. All of the data are expressed as mean ± SEM and processed with Graphpad software.

## Results

### Bioinformatics analysis of CyPs

**Figure 1:**
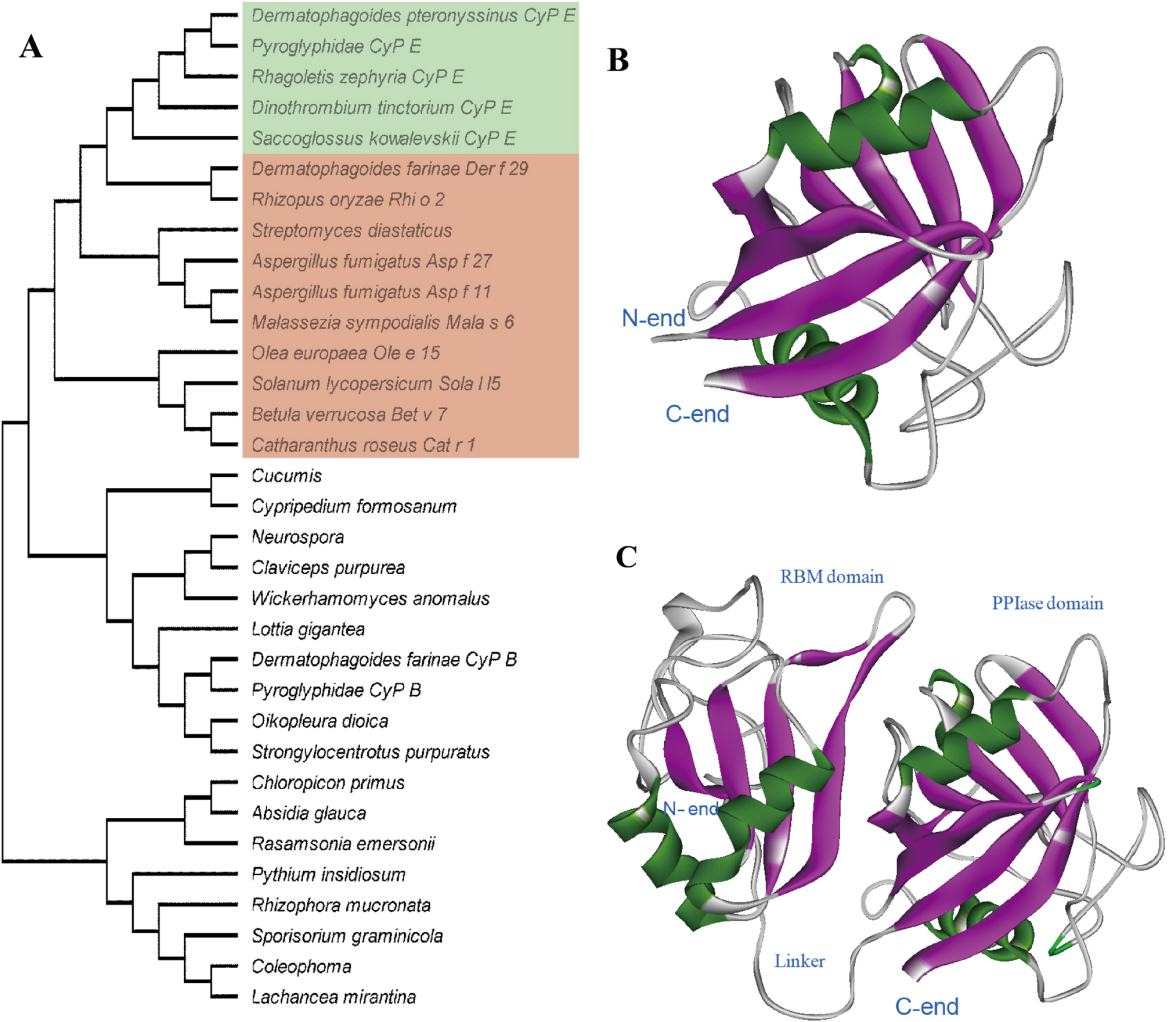
Phylogenetic analysis and three-dimensional structure model of CyPs. (**A**) Evolutionary tree of CyPs. Allergens CyPs are highlighted by red background and CyPE are highlighted by green background. (**B**) Three-dimensional structure model of CyPA. (**C**) Three-dimensional structure model of CyPE.

CyPs proteins are a well-known pan-allergen and belong to the cyclophilin family. The feature and diversity of CyPs family allergens are not reported. In this paper, thirty-three protein sequences of the CyPs family were obtained from the database of NCBI and UniRef to construct an evolutionary tree (Fig. 1A). The identified allergens in thirty-three protein sequences almost belong to CyPA, such as Der f 29, Asp f 27, Asp f 11, Mala s 6, Rhi o 2, Bet v 7, Cat r 1, Ole e 15, and Sola l l5, highlighting in the evolutionary tree through using a red background (Fig. 1A). Immuno-positive rate, molecular weight, amino acid composition, theoretical pIs, instability indexes, and GRAVY of those identified allergens were summarized and analyzed based on the data from website of ProtParam and Allergen Nomenclature. CyPE of HDM (GenBank ID: XP_027199043.1) consists of a CyPE-A domain and a RNA-binding motif domain. CyPE-A domain of CyPE encoded 165 amino acid residues with a predicted molecular weight of 18.4 kDa and a theoretical isoelectric point (pI) of 9.35 (Table 1). ProtParam analysis results showed that the physicochemical characteristics of CyP-A domain of CyPE was similar with CyPA. Theoretical pIs, instability indexes, and GRAVY of CyPA and CyP-A domain of CyPE are 7.75–9.35, 11.58–23.57, and -0.455 to -0.07, respectively (Table 1). Phylogenetic analysis illustrated that the CyPE sub-family that was highlighted by a green background in the Fig. 1A had a closer evolutionary relationship with CyPA. These results illustrate that CyPE of HDM maybe a novel allergen.

**Table 1:** Features of CyPs.

Allergen	Immuno- positive rate	Domain	Number of amino acids	Molecular weight (kDa)	Theoretical pI	GRAVY	Instability index
CyPE-A	–	CyP-A	165	18.4	9.35	− 0.432	17.26
Der f 29	35/41 (85%)	CyP-A	164	17.7	9.17	− 0.209	21.26
Asp f 11	27/30 (90%)	CyP-A	178	19.5	8.87	− 0.455	23.57
Asp f 27	30/40 (75%)	CyP-A	163	17.7	7.75	− 0.250	11.58
Mala s 6	20/97 (21%)	CyP-A	162	17.2	8.79	− 0.083	18.08
Rhi o 2	74/114(65%)	CyP-A	164	17.8	8.38	− 0.321	19.33
Bet v 7	(20.8%)	CyP-A	173	18.3	8.68	− 0.234	15.24
Cat r 1	5/15 (33%)	CyP-A	172	18.3	8.36	− 0.270	21.27
Ole e 15	17/79 (22%)	CyP-A	172	18.1	8.68	− 0.070	14.32
Sola l l5	–	CyP-A	171	17.9	8.83	− 0.109	12.21
CyPB	0/10 (0)	CyP-B	224	24.8	7.66	− 0.308	23.60

### Allergenicity analysis of CyPs

The three-dimensional structure of the CyPE and CyPA of HDM was constructed (Fig. 1B and C). The model structure quality of CyPE and CyPA was assessed by PROCHECK analysis, ERRAT program, VERIFY 3D program, ProSa analysis, and QMEAN analysis (Table 2). As shown in Fig. 1B and C, CyPE and CyPA both contain a PPIase domain. CyPE consists of two domains (a RNA-binding motif and a CyP-A domain), which is linked by a flexible 69-amino-acid-long linker. The overall folding of RNA-binding motif (RBM) domain is a classical α-β sandwich structure, a β-α-β-β-α-β topology fold, which is demonstrated by the first structure (Fig. 1C). The three-dimensional structure is a contribution for analyzing the allergenicity mechanism of CyPs.

**Table 2:** Parameters used for structural assessment of CyPE protein.

Protein	Structural assessment methods	Ramachandran plot (%)	*G*-factor	*Z*-score	*Q* value
CyPE	PROCHECK analysis	89.8^E^	− 0.10^I^		
		9.2^F^	0.21^J^		
		1.0^G^	− 0.05^K^		
		0.0^H^			
	ProSa			− 7.73	
	QMEAN				0.88 ± 0.05
5yzg.1	PROCHECK analysis	88.2^E^	− 0.22^I^		
		11.3^F^	0.62^ J^		
		0.5^G^	0.16^ K^		
		0.0^H^			
	ProSa			− 8.08	
	QMEAN				0.79 ± 0.05

^E^Residues in favorable regions; ^F^residues in allowed regions; ^G^residues in generally allowed regions; ^H^residues in disallowed regions;

^I^*G*-factor score of the dihedral bonds; ^J^ *G*-factor score of the covalent bonds; ^K^overall *G*-factor score.

**Figure 2:**
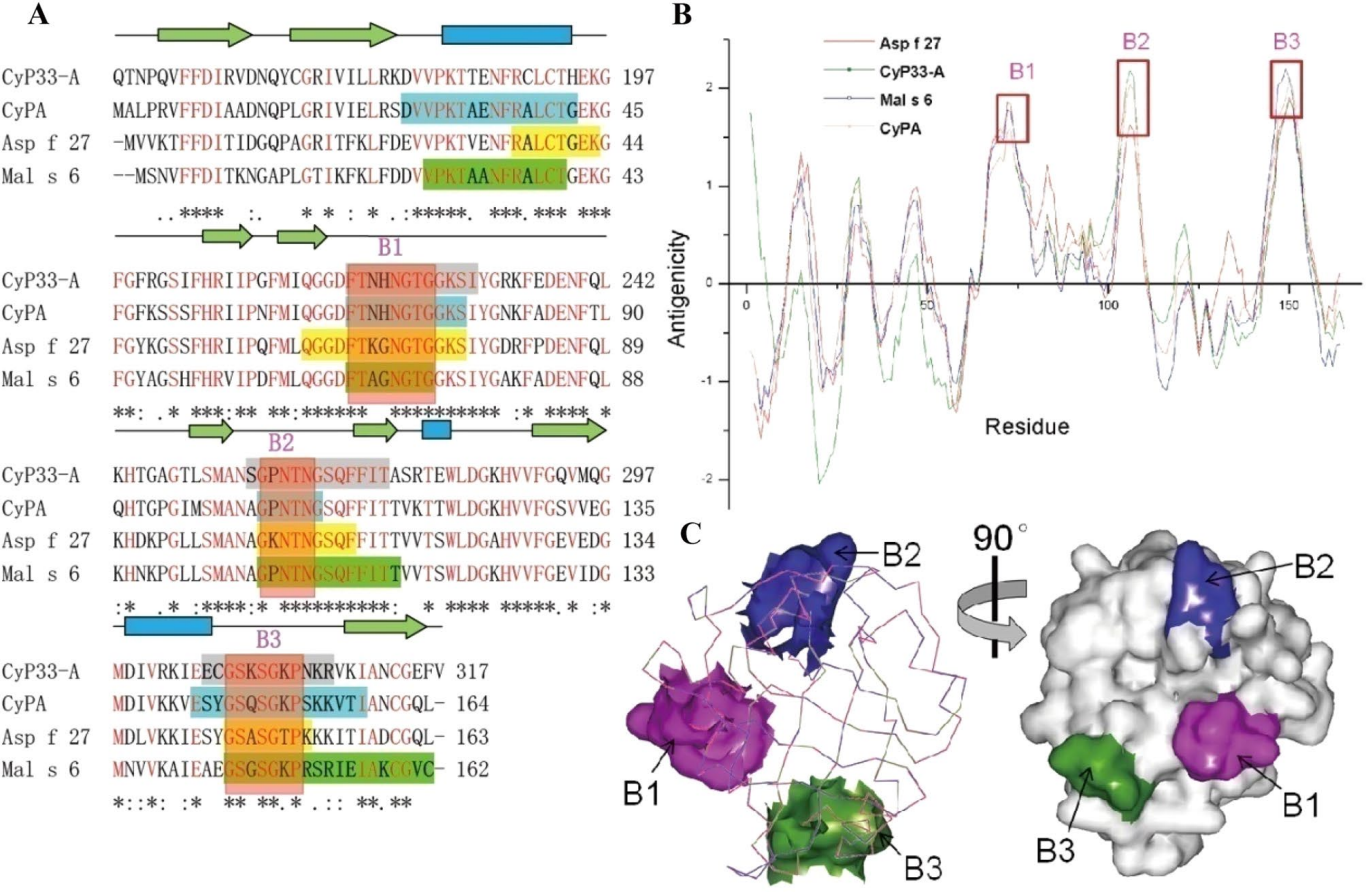
B-cell epitopes and antigenicity of allergen CyPs. (**A**) B-cell epitopes were labeled in sequence alignment of CyPE (CyP33) and other different species CyPs allergens. Sequence alignment of different CyPs performed by multiple alignment. 2D elements were depicted as blue barrels (α-helices) and green arrows (β-sheets). Sequences of B-cell epitope were framed in different colors and named B1–B3. The common epitopes of CyPs were highlighted by a red background. The conserved residues of the four proteins are highlighted by a red font. (**B**) BcePred’s antigenicity plots of the allergen CyPs. B-cell epitopes were framed in a red box and labeled by B1–B3. (**C**) B-cell epitopes B1-B3 are labeled on the superimposed α-carbon backbones structure of CyPs.

DNAStar, BepiPred, and Bcepred systems were used to predict B-cell epitopes (Table 3). The consensus epitopes of the predicted results of the four tools were considered as the ultimate predicting results of B-cell epitopes of CyPs (Table 4). As shown in Fig. 2A, the final B-cell epitopes of CyPs were labeled in the sequence alignment of different species CyPs. The four B-cell epitopes of Der f 29, Asp f 27, and Mala s 6 were predicted. However, the CyPE (CyP33) had only three predicted B-cell epitopes. BcePred’s antigenicity analysis showed that the antigenicity is low at RBM-end of CyPE (Fig. 2B). The immunogenicity of CyPE might be reduced by the RBM of CyPE. The consensus B-cell epitopes of CyPs were showed in the red boxes of Fig. 2A and named as B1, B2, and B3. The structural α-carbon backbone of CyPE’ CyP-A domain superimposed with CyPA. The consensus B-cell epitopes were labeled on the superimposed α-carbon backbone structure the CyPs (Fig. 2C).

**Table 3:** The results of B cell epitopes predictions of CyPs.

	Tools	Location of the prediction results
B cell epitope prediction	DNAStar protean	27–38, 67–82, 107–111, 126–128,152–161
	Cbtope	67–80, 103–115, 125–128,134–165
CyPE	BepiPred	29–33, 66–85, 95–109, 119–122, 144–155
	Bcepred	25–34, 67–84, 99–109, 103–115, 125–128,139–153
B cell epitope prediction	DNAStar protean	10–17, 22–37, 40–52, 62–88, 103–111, 121–125,137–157
	Cbtope	27–43, 67–82, 107–111, 123–129,142–163
Der f 29	BepiPred	12–17,28–33,45–48, 66–84,89–93,102–111, 143–157
	Bcepred	23–34,39–51, 63–77, 102–109, 126–132,139–162
B cell epitope prediction	DNAStar protean	8–15,20–32, 38–45, 60–77, 80–95, 101–109, 120–124,140–161
	Cbtope	27–39, 65–72, 91–97, 105–115, 145–162
Mala s 6	BepiPred	10–15,27–39,42–46, 64–76, 91–115, 141–152
	Bcepred	24–42, 59–74, 87–97, 100–115, 140–151
B cell epitope prediction	DNAStar protean	10–18, 27–51, 62–74, 77–96, 101–110, 130–157
	Cbtope	30–43, 61–78, 88–94, 106–111, 145–151
Asp f 27	BepiPred	11–18, 36–48, 65–94, 102–111, 143–154
	Bcepred	36–50, 61–76, 88–94, 100–107, 142–152

**Table 4:** Predicted B-cell epitope sequences of allergen CyPs.

Allergen	Position	Sequence
CyPE	–	–
	67–78	FTNHNGTGGKSI
	103–115	SGPNTNGSQFFIT
	144–155	ECGSKSGKPNKR
Mal s 6	36–43	RALCTGEK
	62–76	QGGDFTKGNGTGGKS
	88–94	QLKHDKP
	145–151	GSASGTP
Der f 29	27–43	DVVPKTAENFRCLCTG
	67–77	FTNHNGTGGKS
	107–111	TNGSQ
	143–157	ESYGSQSGKPSKKVTI
Asp f 27	27–39	DVVPKTAENFRCLCTG
	65–72	FTAGNGTG
	91–97	NKPGLLS
	145–162	SGSGKPRSRIEIAKCGVC

### Allergenic analysis of HDM CyPs

**Figure 3:**
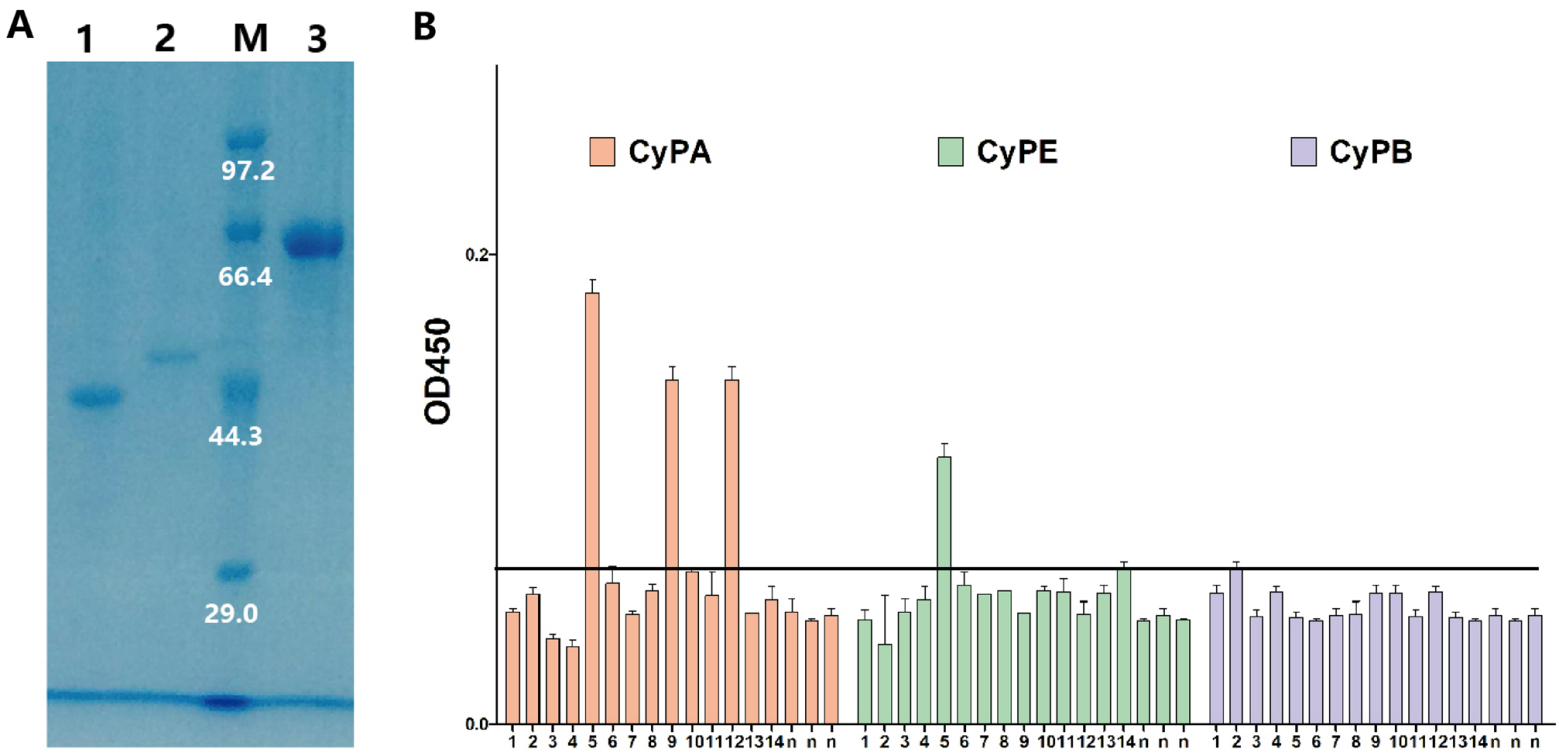
Expression and IgE activities of CyPs. (**A**) SDS-PAGE analysis of the purified CyPs. Lane M: Protein Marker. Lane 1: the purified CyPA; Lane 2: the purified CyPB; Lane 3: the purified CyPE. (**B**) The specific IgE reactivity to allergen CyPs by ELISA. n, the serum from healthy subjects (n = 3); P1-P14, the serum from the patients with dust-mite allergy.

To verify the results of in silico prediction, CyPA, CyPE, and CyPB of HDM was obtained. The pET32a^+^ expression vector was used to express the recombinant CyPs proteins. The purified CyPs (CyPA, CyPE, and CyPB) were examined by SDS-PAGE (Fig. 3A), demonstrating that the apparent molecular weight of CyPA, CyPB, and CyPE was about 40.1 kDa, 47.6 kDa, and 59.2 kDa, respectively. The pET32a^+^ expression vector embodied several protein Tags that were co-expressed with target protein to improve solubility and purification. The molecular weight of the protein Tags was about 21.0 kDa including one Trx-Tag, one S-Tag, two His-Tags and protein linker. The ExPASy online tool Compute pI/Mw was used to predict the theoretical weight of CyPs proteins (https://web.expasy.org/compute_pi/). The results illustrated that the theoretical weights of CyPA (17.8 kDa), CyPB (24.8 kDa), and CyPE (35.4 kDa) were nearly congruent with those of apparent molecular weight subtracting protein Tags of co-expression. The dichroic spectra (Fig. S2) showed no obvious difference in the secondary structure of recombinant CyPs proteins (CyPA, CyPB and CyPE). ELISA was performed using serums from 26 dust-mite allergic patients. ELISA results indicated that 3/14, 1/14 and 0/14 patient serum had positive reactions to CyPA, CyPE and CyPB, respectively. The ELISA value of CyPA was more than twice the value of CyPE. CyPB did not show positive reactions of patient serum.

## Discussion

Cyclophilins (CyPs) participate in various physiology and pathology, such as signal transduction, cell proliferation, cell necrosis, bacteria and parasitic disease^47,48^. CyPs, a well-known pan-allergen, are identified with five classic CyP isoforms (CyPA, B, C, D and E). CyPA of HDM was reported as a main allergen CyPA^49^. In addition, HDM are a key allergen in the room^50^. Therefore, research on immune response and characterization of structure and epitopes of HDM CyPs family will be beneficial for the diagnosis and treatment of allergic disease.

In order to elucidate the allergic mechanism of CyPs, the physicochemical and immunologic characteristics of several CyPs were analyzed via bioinformatics approaches and allergenic databases. The results showed that CyPE had a close evolutionary relationship and a high sequence identity with allergenic CyPA (Fig. 1A). And the PPlase domain of CyPE was superimposed with allergenic CyPA in structural α-carbon backbones (Fig. 2C). The surface receptors of B-cells and T-cells can recognize epitopes of allergens and activate immune cells^51^. The predicted B-cell epitopes of CyPE’ CyP-A domain demonstrated the sharing of consensus epitopes with CyPA. The structure and antigenicity analysis showed that CyPE-A domain of CyPE conserved antigenic surfaces and solvent-accessibility of their putative epitopes compared with CyPA (Fig. 2).

**Figure 4:**
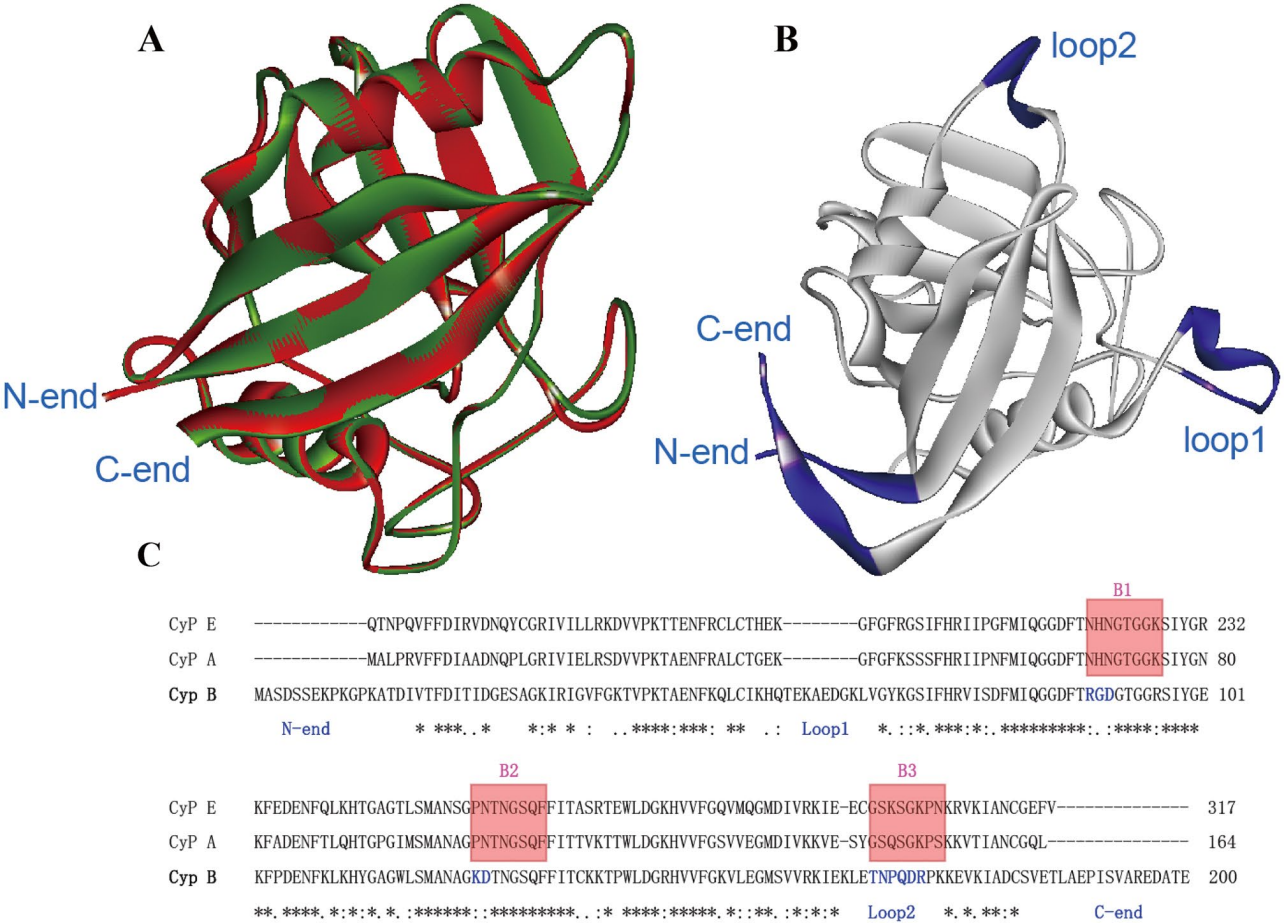
Structure and sequence analysis of allergen CyPs (CyPA and CyP33-A) and no-allergen CyPs (CyPB). (**A**) The superimposed structure of allergen CyPs (CyPA and CyP33-A). (**B**) The structure of no-allergen CyPs (CyPB). (**C**) Sequence analysis of allergen CyPs (CyPA and CyP33-A) and no-allergen CyPs (CyPB). The consensus epitopes of CyPs were highlighted by a red background, and the related residues of no-allergen CyPs are marked by a blue font.

In order to verify in silico results, CyPA, CyPE and CyPB from HMD were successfully purified. ELISA results indicated that the positive reaction rates of CyPs to patient serum are CyPA (21.4%), CyPE (7.1%) and CyPB (0%), respectively, illustrating that the IgE activity was exhibited in CypA and CypE excluding CyPB. BcePred’s antigenicity analysis showed that CyPE is low antigenicity at RBM-end (Fig. 2B). The RBM domain of CyPE may reduce the immunogenicity of CyPE. In addition, we observe that CyPB showed a further evolutionary relationship with allergenic CyPA than CyPE in the evolutionary tree (Fig. 1A). The whole structure of CyPB is similar to CyPA, except for peptides of loop1, loop2, C-end, and N-end (Fig. 4A and B), which is consistent with its reported in the two loop regions (residues 19–24 and 152–164) and at the amino and carboxyl termini^52^. We recognize that CyPB has significant differences from allergen CyPs at B1-B3 sequence of B-cell epitopes. The amino acid sequence (N–H–N) of B1 was replaced by R-G-D of CyPB and the amino acid sequence (P–N) of B2 was replaced by K-D of CyPB. Amino acid sequence of B3 was replaced by whole loop2 (Fig. 4C). That may be the reason that CyPB has no IgE activity^53^.

In summary, bioinformatics and immunoinformatics analysis illustrate that CyPE is an essential element of cyclophilin allergen family. CyPA and CyPE both have are the IgE activity and CyPB have no IgE activity. Three consensus B-cell epitopes of CyPE and CyPA are determined ultimately by multiple immune information tools. ELISA results indicated that the positive reaction rates of CyPs to patient serum are CyPA (21.4%), CyPE (7.1%) and CyPB (0%), illustrating that the IgE activity was exhibited in CypA and CypE excluding CyPB. Structure and immunoinformatics analysis demonstrated that the RNA-binding motif (RBM) of CyPE could reduce the immunogenicity of CyPE. The reason that CyPB has no IgE activity might be the structure mutation of CyPB on B-cell epitopes.

### Supplementary Information

Supplementary Information.

## Data Availability

The datasets used and/or analysed during the current study available from the corresponding author on reasonable request.
