# Supplementary material for: Structure/epitope analysis and IgE binding activities of three cyclophilin family proteins from Dermatophagoides pteronyssinus
Source: Sci Rep. 2023 Aug 21;13:13630. doi: 10.1038/s41598-023-40720-6 (PMC10442367; doi:10.1038/s41598-023-40720-6)
Supplement: Supplementary file 1 — Supplementary Information. [file 41598_2023_40720_MOESM1_ESM.doc]

Structure/epitope analysis and IgE binding activities of three cyclophilin family proteins from *Dermatophagoides pteronyssinus*

Yuwei Li^1,2,3^, Xizhuo Sun^2^, and Liteng Yang^1,2*^

^1^ Department of Respiratory Medicine. The seventh affiliated hospital of Southern Medical University, Foshan, Guangdong 528244, China.

^2^ Department of Respirology & Allergy. The Third Affiliated Hospital of Shenzhen University, Shenzhen 518020, China.

^3^ Laboratory of Chemical Biology and State Key Laboratory of Rare Earth Resource Utilization, Changchun Institute of Applied Chemistry, Chinese Academy of Sciences, Changchun, Jilin 130022, China.


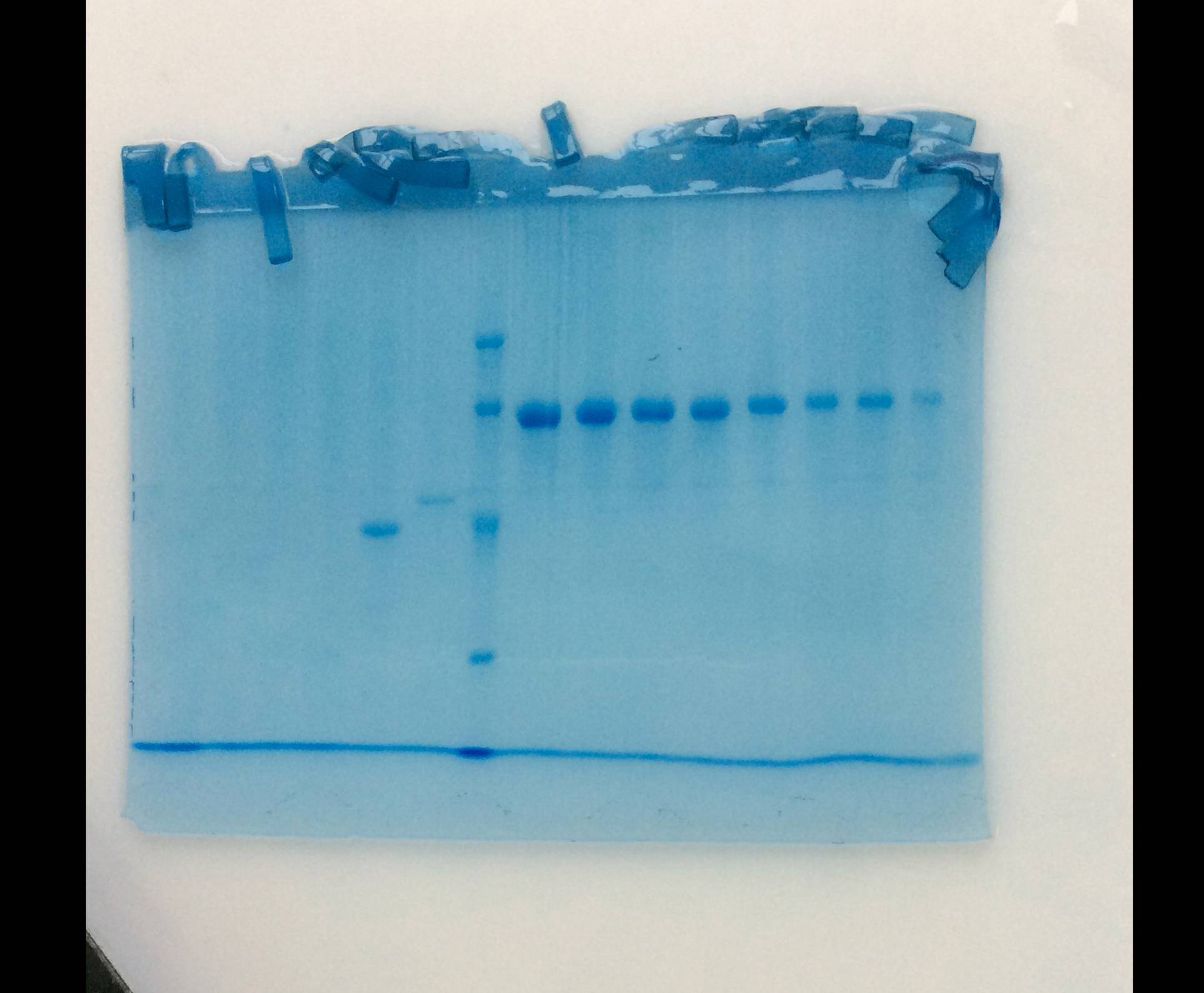


**Fig. S1.** SDS-PAGE analysis of the purified CyPs (CyPA, CyPB and CyPE). Left lane of the marker lane: the purified CyPA and CyPB; Right lane of the marker lane: the loading different amounts of CyPE.


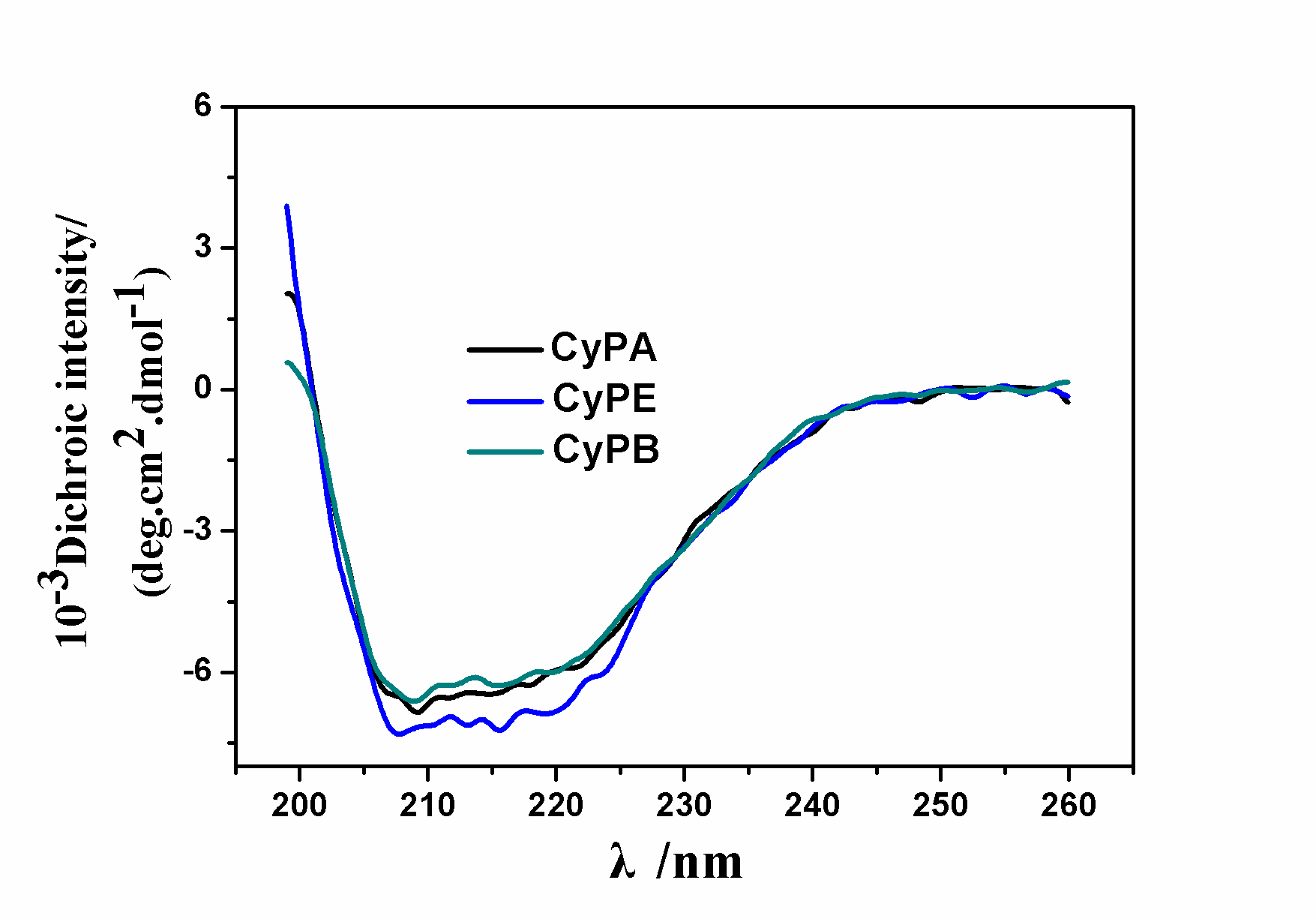


**Fig. S2.** The dichroic spectra of recombinant CyPs proteins (CyPA, CyPB and CyPE).
